# Navarixin alleviates cardiac remodeling after myocardial infarction by decreasing neutrophil infiltration and the inflammatory response

**DOI:** 10.3389/fphar.2025.1535703

**Published:** 2025-03-13

**Authors:** Peikun Hu, Wei Liu, Jungang Huang, Yangfan Su, Huiqi Jiang, Qinyu Wu, Jun Tao, Shi Liang, Jun Lin, Junmeng Zheng

**Affiliations:** ^1^ Department of Cardiovascular Surgery, Sun Yat-sen Memorial Hospital, Sun Yat-sen University, Guangzhou, China; ^2^ Department of Nephrology, The Third Affiliated Hospital, Southern Medical University, Guangzhou, China

**Keywords:** myocardial infarction, navarixin, neutrophils, inflammatory response, fibrosis, transcriptomics

## Abstract

Coronary atherosclerotic heart disease is an important, worldwide burden on human health. Central muscle infarction is the most dangerous condition, has the highest mortality and disability rates, and is gradually becoming more common among young people. After myocardial infarction, neutrophils recruited to the infarcted area promote the myocardial inflammatory response by releasing proinflammatory factors and chemokines and release matrix metalloproteinases and myeloperoxidases that degrade the extracellular matrix and produce reactive oxygen species, resulting in irreversible myocardial damage and thereby promoting ventricular remodeling. In this study, we constructed a mouse model of myocardial infarction and utilized the CXCR2 receptor inhibitor navarixin (Nav) to reduce neutrophil recruitment after MI. We observed that Nav improved cardiac function, reduced myocardial damage, reduced neutrophil infiltration, reduced inflammatory factor expression and improved cardiac fibrosis in mice. Through transcriptomic analysis, we found that Nav affects signaling pathways such as the innate immune response and the chemokine signaling pathway, thereby decreasing the inflammatory response by reducing neutrophil chemotaxis. This study provides new insights for the use of CXCR2 inhibitors as new therapeutic options for myocardial infarction in the future.

## 1 Introduction

Coronary atherosclerotic heart disease (CHD), a common heart disease worldwide, is an important burden on human health. Among these conditions, myocardial infarction (MI) is the most dangerous, has the highest mortality and disability rates, and is gradually becoming more common among younger people (Lindahl and Mills, 2023). High blood pressure, diabetes, smoking, and excessive alcohol consumption are all risk factors for CHD (Damluji et al., 2021; Gong et al., 2021). Although therapeutic drugs and interventional treatments have greatly reduced the mortality rate of myocardial infarction, the rapid reduction and/or interruption of coronary blood flow after myocardial infarction causes severe ischemia and hypoxia in the myocardial tissue supplied by the artery, resulting in a large amount of myocardial cell necrosis and eventually causing serious consequences such as heart failure (Frantz et al., 2022; Rallidis et al., 2022).

These necrotic myocardial tissues promote the initiation of the immune inflammatory response, and many immune cells accumulate in the infarct area to remove necrotic cells (Vafadarnejad et al., 2020; Daseke et al., 2021). As the main cell type involved in the acute inflammatory response, neutrophils are recruited to the infarct area at an early stage to promote the myocardial inflammatory response by releasing proinflammatory factors and chemokines and by releasing matrix metalloproteinases and myeloperoxidases to degrade the extracellular matrix and to produce reactive oxygen species, resulting in irreversible myocardial damage and thus promoting ventricular remodeling (Ogura et al., 2021; Zhou et al., 2022). Controlling the recruitment of neutrophils to the infarcted myocardium can effectively reduce the release of inflammatory factors, thereby reducing myocardial inflammation (Sreejit et al., 2020; Jiang et al., 2022). Therefore, neutrophils play a nonnegligible role in the pathophysiological process of ventricular remodeling after myocardial infarction, and the regulation of neutrophil function is very important for myocardial infarction injury repair.

Chemokines are a family of small cytokines that can induce the directed migration and activation of leukocytes. The biological functions of chemokines are achieved by binding to cell surface receptors, which are divided into four broad categories based on their N-terminal cysteine residues: CCRs, CXCRs, CX3CRs, and XCRs. Accumulating evidence suggests that these chemokines play an important role in promoting the development of inflammatory responses by recruiting immune cells such as neutrophils to the injured myocardium. CXCR2 is a 360-amino acid glycoprotein expressed in many types of cells and tissues, and CXCR2 synergistically binds to its ligands CXCL1-3 and CXCL5-8 to control the release of neutrophils from the bone marrow and recruit them to inflamed and infected tissues. Studies have shown that CXCR2 plays an important role in promoting the migration and recruitment of monocytes/macrophages to damaged heart and arterial walls and promoting pathological myocardial remodeling, acute myocardial infarction and atherosclerosis.

Navarixin is an allosteric, noncompetitive, and orally active antagonist of CXCR2. In recent years, numerous studies have shown that navarixin can alleviate the occurrence and development of diseases such as tumors, calcifications, infections, and trauma by antagonizing CXCR2 (Gonsiorek et al., 2007; Nair et al., 2012; Kredel et al., 2014; Russo et al., 2014; Jones and Levy, 2018). Clinically, the compound is being evaluated in four phase II trials for the treatment of psoriasis, cancer, and asthma; while promising *in vivo* and clinical trial results have been published, larger, longer-lasting studies are needed (Holz et al., 2010; Hastrup et al., 2015; Rennard et al., 2015; Todd et al., 2016; Ashar et al., 2021; Chabry et al., 2023; Dahal et al., 2023; Dhayni et al., 2023). However, there have been no studies on navarixin in the field of cardiovascular diseases, especially myocardial infarction. In this study, we treated mice with myocardial infarction with navarixin to observe whether it could inhibit the recruitment of neutrophils after myocardial infarction and the subsequent increase in the inflammatory response, tested its ability to alleviate adverse injury repair and fibrosis mechanisms after myocardial infarction, and explored the related mechanisms by transcriptomics.

## 2 Materials and methods

### 2.1 Mice and treatments

Wild-type C57BL/6 male mice (8 weeks old, 18–22 g body weight) were randomly divided into three groups: (1) the sham operation group (n = 5), (2) the myocardial infarction control group (saline, oral dosage, once daily for 4 weeks after MI operation) (n = 5), and (3) the myocardial infarction-navarixin treatment group (1 mg/kg, oral dosage, once daily for 4 weeks after MI operation) (n = 5). The number of mice in each group was five. Mice were housed under a constant 12 h light/dark cycle. The mice were housed at an appropriate temperature and humidity, and all the mice had free access to food and water. Navarixin was purchased from MedChemExpress (MCE) Company (HY-10198). All animal experiments were approved by the Animal Ethical and Welfare Committee, SYSMH. (approval number: AP20230093; Certification date: 20 June 2023).

### 2.2 Myocardial infarction model

Eight-week-old male wild-type mice were anesthetized with 2% isoflurane and intubated with a small rodent ventilator. After left thoracotomy in the fourth intercostal space, 8–0 line ligation of the anterior descending branch of the left coronary artery was used to induce myocardial infarction. In the sham surgical group, the artery was not ligated, but the other procedures were the same. The thoracic incision was then sutured, and the mouse was placed on a heating pad for rehabilitation.

### 2.3 Echocardiography

To assess left ventricular functionality, the mice were anesthetized with 2% isoflurane, and echocardiography was performed using a Vevo 2100 ultrasound system. The ejection fraction, fractional shortening, and ventricular chamber dimensions were measured using the parasternal short-axis (M-mode) view to determine the ejection fraction (EF), fractional shortening (FS), end-systolic diameter (ESD), end-diastolic diameter (EDD), end-systolic volume (ESV), and end-diastolic volume (EDV). Heart rate was monitored throughout the imaging session. The left ventricle weight and body weight were obtained at the end of the study to calculate the left ventricular-to-body weight ratio (LV/BW).

### 2.4 Masson staining

After the heart samples were harvested, they were fixed with paraformaldehyde, dehydrated and embedded in paraffin. The heart was cross-cut with a paraffin microtome with a cross-sectional thickness of 5 μm. Staining was performed using Masson’s tricolor staining protocol, and images were captured under a light microscope for subsequent analysis. ImageJ software (NIH, Maryland, United States) was used to divide the blue, i.e., fibrotic area, by the total myocardial area to obtain the percentage of the fibrotic area.

### 2.5 Western blotting assay

The left ventricles of mice from each group were collected, and protein extracts were prepared using RIPA lysis buffer. The protein concentration in each group of samples was determined by the BCA Protein Concentration Assay Kit. Protein electrophoresis was performed on an 8% SDS‒PAGE gel, and the proteins were transferred to a PVDF membrane (Cat# 88518, Thermo Fisher). The PVDF membrane was blocked with 5% skim milk for 1 h at room temperature. After blocking, the membrane was incubated overnight with primary antibodies at 4°C. The primary antibodies used were as follows: anti-fibronectin, anti-collagen 1, anti-α-SMA, and anti-GAPDH. The next day, the membranes were incubated with secondary antibodies for 2 h at room temperature, and protein bands were detected with enhanced chemiluminescence (ECL) and visualized with a gel imager.

### 2.6 Real-time PCR

We extracted RNA from macrophages isolated from the left ventricular tissue of mice in various groups. RNA extraction using TRIzol reagent was performed following the manufacturer’s instructions. QRT Mix was used for reverse transcription of the qPCR products to cDNA. RT‒PCR analysis with a LightCycler 480 detector was used to measure the gene expression levels of inflammatory factors (IL-1β, IL-6, CXCR1, and CXCR2). The results are shown as cycle thresholds (Ct). mRNA expression levels were quantified using GAPDH as an mRNA control and compared using the 2^−ΔΔCT^ comparison method.

### 2.7 Measurement of myocardial injury markers

The qualified mouse serum samples were obtained by 10 min centrifugation of collected blood specimens. For the assessment of myocardial enzyme leakage, the activity of serum creatine kinase (CK), the MB CK isoenzyme (CK-MB) and cardiac troponin (cTnT) were assessed according to the corresponding kit manufacturers’ instructions.

### 2.8 Flow cytometry

The left ventricles (LVs) of mice from each group were collected 7 days after myocardial infarction. The LVs were rinsed with PBS, and the LVs were digested in collagenase II solution. Red blood cells were removed using red blood cell lysis buffer, and the cells were blocked with blocking buffer and incubated with anti-mouse CD45, CD11b, F4/80 and Ly6G antibodies in the dark at 4°C. Then, the cells were washed in PBS and subjected to flow cytometry or cell sorting by flow cytometry. The results were analyzed using FlowJo software.

### 2.9 Transcriptomic analysis

#### 2.9.1 RNA extraction, library construction and sequencing

Total RNA was extracted using TRIzol reagent following the manufacturer’s instructions. The total RNA quantity and purity were analyzed with a Bioanalyzer 2100 and RNA 6000 Nano LabChip Kit, and high-quality RNA samples with an RNA integrity number (RIN) > 7.0 were used to construct a sequencing library. After total RNA was extracted, mRNA was purified from total RNA (5 µg) using Dynabeads Oligo (dT) with two rounds of purification. Following purification, the mRNA was fragmented into short fragments using divalent cations at elevated temperature (magnesium RNA fragmentation module at 94°C for 5–7 min). Then, the cleaved RNA fragments were reverse-transcribed to create cDNA by SuperScript™ II Reverse Transcriptase, which was subsequently used to synthesize U-labeled second-strand DNAs with *E. coli* DNA polymerase I, RNase H (NEB, cat. m0297, United States) and dUTP Solution. An A-base was then added to the blunt ends of each strand, preparing them for ligation to the indexed adapters. Each adapter contained a T-base overhang for ligating the adapter to the A-tailed fragmented DNA. Dual-index adapters were ligated to the fragments, and size selection was performed with AMPureXP beads. After heat-labile UDG enzyme treatment of the U-labeled second-strand DNAs, the ligated products were amplified via PCR under the following conditions: initial denaturation at 95°C for 3 min; eight cycles of denaturation at 98°C for 15 s, annealing at 60°C for 15 s, and extension at 72°C for 30 s; and a final extension at 72°C for 5 min. The average insert size for the final cDNA libraries was 300 ± 50 bp. Finally, we performed 2 × 150 bp paired-end sequencing (PE150) on an Illumina NovaSeq™ 6,000 following the vendor’s recommended protocol.

#### 2.9.2 Differentially expressed gene (DEG) analysis

Gene differential expression analysis was performed by DESeq2 software between two different groups (and by edgeR between two samples). Genes with a false discovery rate (FDR) less than 0.05 and an absolute fold change ≥2 were considered differentially expressed genes. Differentially expressed genes were then subjected to enrichment analysis of GO functions and KEGG pathways.

#### 2.9.3 GO enrichment analysis

Gene Ontology (GO) is an international standardized gene functional classification system that offers a dynamically updated controlled vocabulary and a strictly defined concept to comprehensively describe the properties of genes and their products in any organism. GO has three ontologies: molecular functions, cellular components and biological processes. The basic unit of GO is the GO term. Each GO term belongs to a type of ontology. GO enrichment analysis revealed all GO terms that were significantly enriched in DEGs compared to the control genome. First, all DEGs were mapped to GO terms in the Gene Ontology database (http://www.geneontology.org/), gene numbers were calculated for every term, and significantly enriched GO terms in DEGs compared to the control genome were defined by a hypergeometric test.

#### 2.9.4 Pathway enrichment analysis (KEGG)

Genes usually interact with each other to play roles in certain biological functions. Pathway-based analysis helps to further understand the biological functions of genes. KEGG is a major public pathway-related database. Pathway enrichment analysis revealed significantly enriched metabolic pathways or signal transduction pathways in DEGs compared with the whole-genome background.

### 2.10 Statistical analysis

All the data were statistically analyzed using Prism 8.0 (GraphPad software). All the data are expressed as the means ± standard deviations. Significant differences in the means between two groups were determined using Student’s t-test, and one-way analysis of variance (ANOVA) was used to compare differences between groups. A p value of <0.05 was considered to indicate statistical significance. Significance is indicated as follows; NS: non-significant, P ≥ 0.05; *P < 0.05; **P < 0.01; ***P < 0.001.

## 3 Results

### 3.1 Nav improves cardiac function after myocardial infarction

**FIGURE 1:**
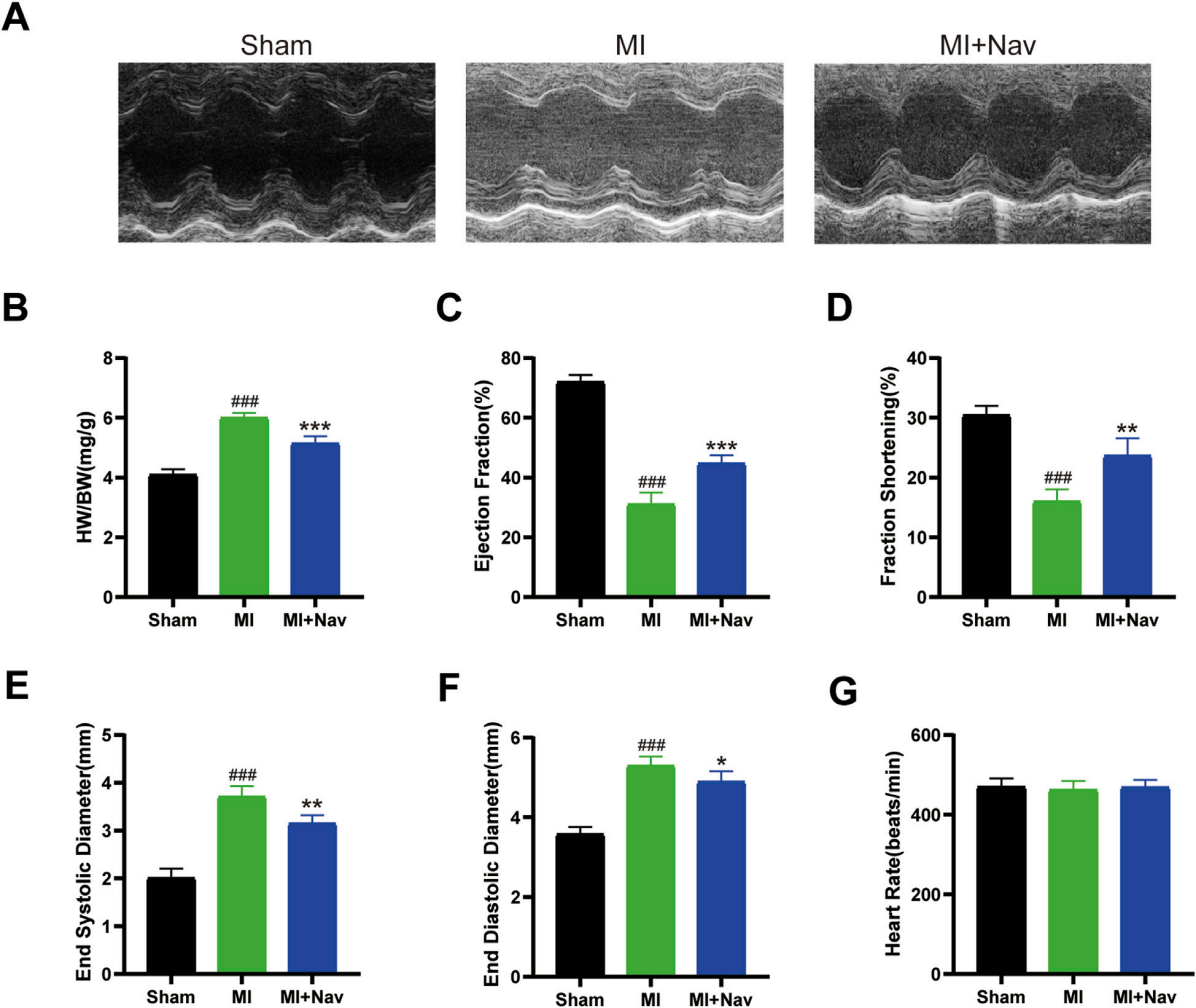
**(A)** Typical M-shaped echocardiogram images. **(B)** The heart weight–to–body weight (HW/BW) ratio in the three groups. (n = 5) **(C**–**F)** Echocardiographic results of the EF, FS, ESD, and EDD. (n = 5) **(G)** Heart rates of the mice in the four groups. (n = 5) #P < 0.05, ##P < 0.01, ###P < 0.001 vs. the sham group; *P < 0.05, **P < 0.01, ***P < 0.001 vs the MI group.

To determine whether the chemokine receptor CXCR2 inhibitor navarixin has a positive effect on cardiac function after MI in mice, we measured and calculated the ejection fraction (EF%) and short-axis shortening rate (FS%) in each group of mice, as shown in Figure 1A. The results showed that the heart rates of the mice in each group were consistent. Compared with those in the sham group, the ejection fraction (EF) and fraction shortening (FS) in the MI group were reduced, but this effect was alleviated by Nav treatment. We also measured the end-systolic diameter (ESD) and end-diastolic diameter (EDD) by echocardiography and found that Nav attenuated MI-induced ventricular enlargement. Moreover, the reduction in the left ventricular weight-to-body weight ratio in the Nav treatment group compared to the control group also demonstrated that Nav can reduce cardiac remodeling caused by MI. In conclusion, the decline in heart function after MI was alleviated after Nav treatment.

### 3.2 Nav treatment limits cardiac injury and alleviates cardiac fibrosis after MI

**FIGURE 2:**
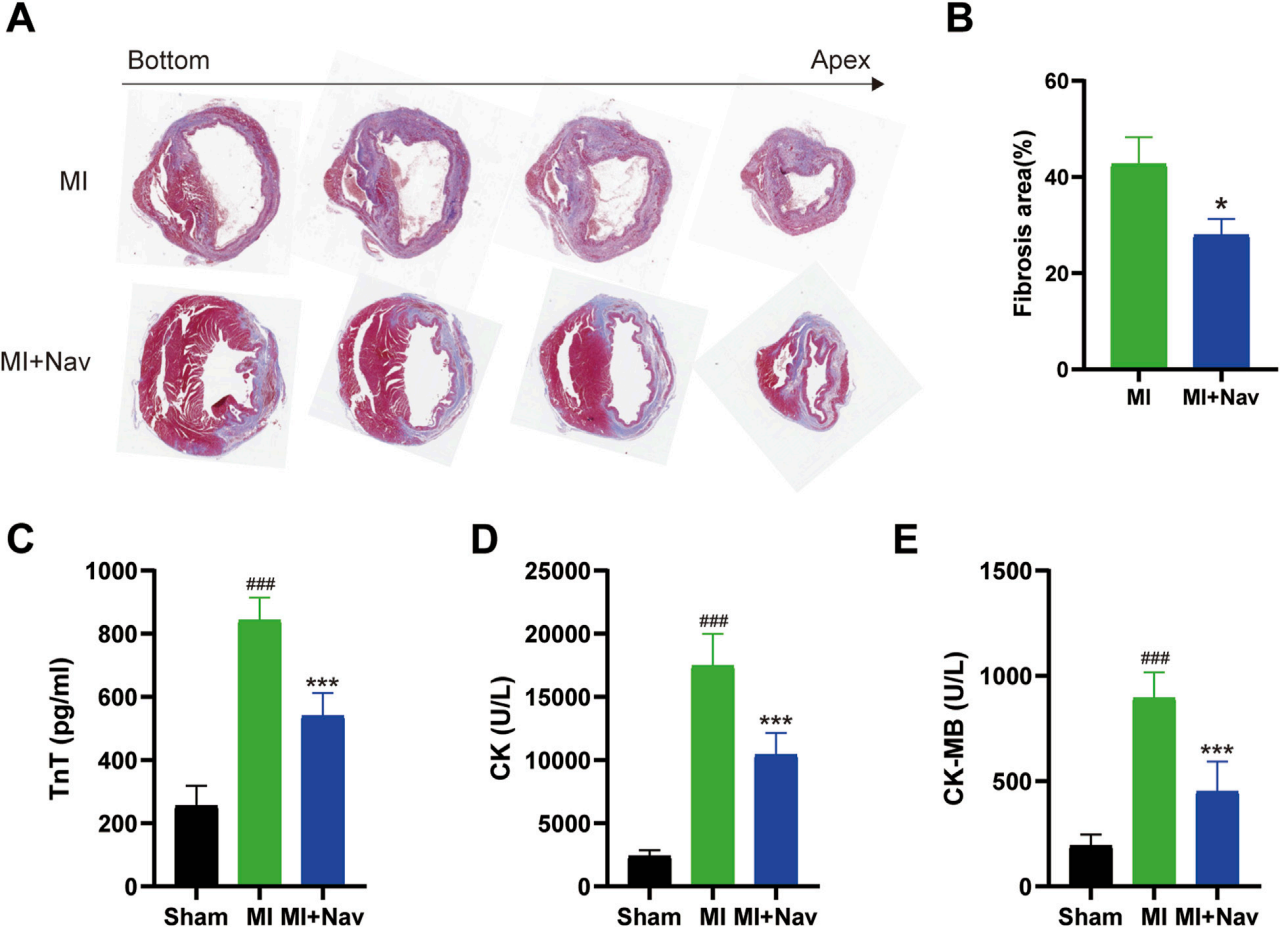
**(A)** Masson’s trichrome staining of the MI and MI + Nav groups. **(B)** Statistical analysis of the fibrosis area fraction. **(C**–**E)** Effects of Nav on serum CK, CK-MB and TnT concentrations. *P < 0.05, **P < 0.01, ***P < 0.001 vs. the MI group.

MI induces myocardial hypertrophy or ventricular remodeling, which is characterized by increased cardiomyocyte volume, interstitial and perivascular fibrosis, loss of cardiomyocytes, increased collagen abundance, and myofibroblast activation. Early myocardial hypertrophy and ventricular remodeling are compensatory responses of the heart to maintain its normal ejection function, and if pathological factors are eliminated in time, they generally do not adversely affect cardiac function. If the pathological factors are not eliminated in time, this series of changes cannot be improved in a short time and this leads to poor myocardial hypertrophy and ventricular remodeling. Eventually, the heart loses its ability to compensate. The consequences of this can be considerable, and in severe cases, they can be life-threatening. To investigate whether Nav has an improvement effect on adverse myocardial fibrosis after myocardial infarction, we performed Masson’s trichrome staining. The results showed that there was a large amount of collagen accumulation in the left ventricular tissue in the MI group, and Nav treatment reduced collagen accumulation (Figures 2A, B). Moreover, after Nav treatment, the heart was able to tolerate MI-induced necrosis, as the levels of cardiac enzymes (including TnT, CK, and CK-MB) were reduced after Nav administration (Figures 2C–E). Therefore, these data suggest that Nav is an effective cardioprotective compound after myocardial infarction.

### 3.3 Nav treatment reduced neutrophil numbers after MI

**FIGURE 3:**
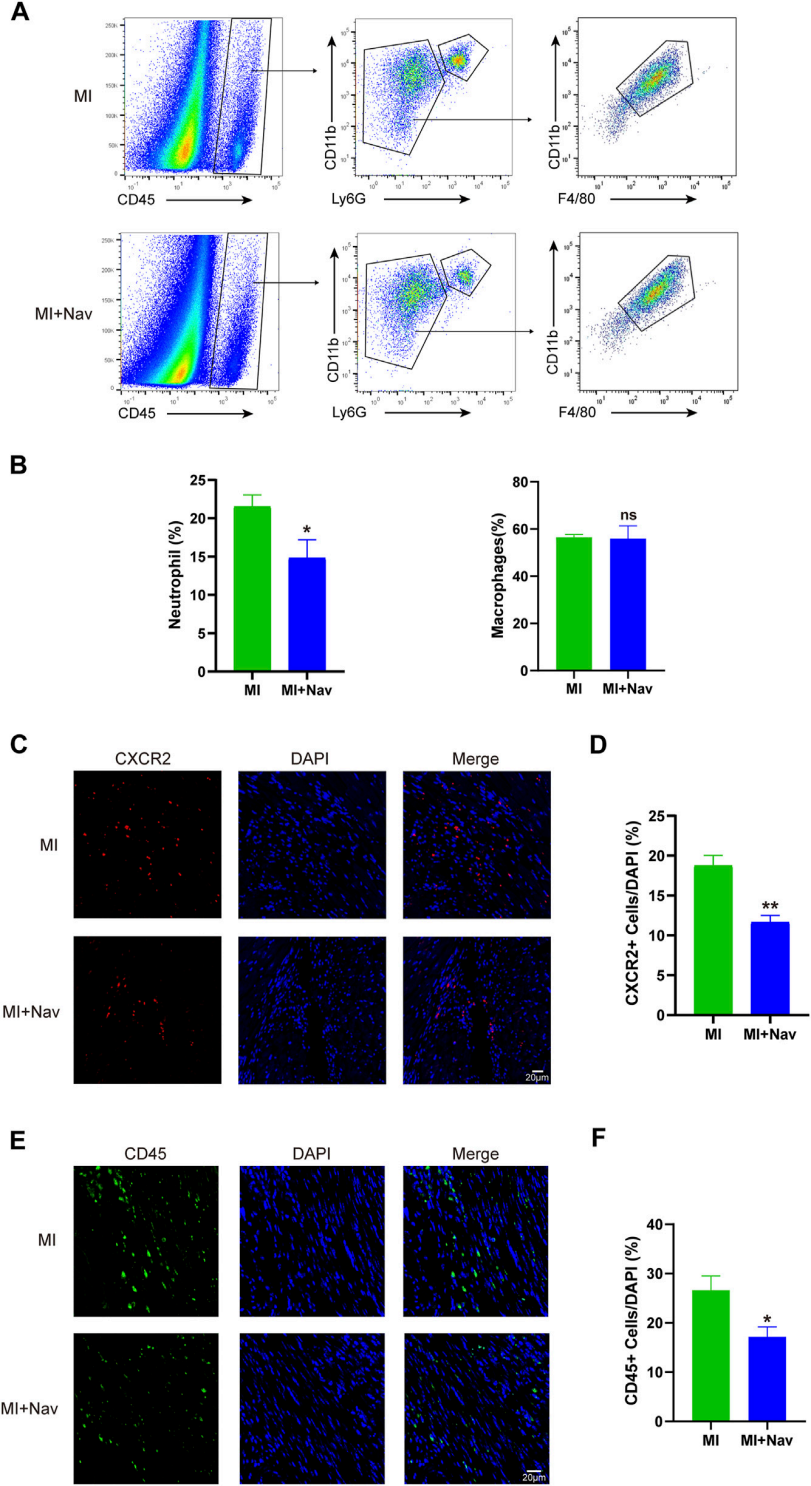
**(A)** Flow cytometry analysis strategies for neutrophils and macrophages. **(B)** Chart of the flow cytometry analysis. **(C)** Typical images of CXCR2 immunofluorescence staining in the two groups. **(D)** Chart of the immunofluorescence staining analysis. **(E)** Typical images of CD45 immunofluorescence staining in the two groups. **(F)** Chart of the immunofluorescence staining analysis. *P < 0.05, **P < 0.01, ***P < 0.001 vs. the MI group.

Neutrophils can promote the inflammatory response after MI. Aggregated activated neutrophils promote the myocardial inflammatory response by releasing proinflammatory factors and chemokines and releasing matrix metalloproteinases and myeloperoxidases that degrade the extracellular matrix and produce reactive oxygen species; this results in irreversible myocardial damage and promotes ventricular remodeling. Therefore, controlling the recruitment of neutrophils to the infarcted myocardium can effectively reduce the release of inflammatory factors, thereby reducing myocardial inflammation. We quantified the proportion of neutrophils after Nav treatment by flow cytometry, first defining CD45^+^ cells as immune cells, from which we sorted neutrophils by CD11b and Ly6G expression. We found that the proportion of neutrophils in the heart was greater after Nav treatment than in the control MI group. Macrophages also play a crucial role in the inflammatory response following MI, and their involvement in the anti-fibrotic and anti-inflammatory effects of Navarixin warrants further clarification. To address this, we assessed macrophage recruitment following Navarixin treatment using flow cytometry, and our results indicate that its effect on macrophage infiltration is negligible (Figures 3A, B). Moreover, we used immunofluorescence to stain the hearts of the two groups for CXCR2 expression and found that the proportion of CXCR + neutrophils in the Nav group decreased significantly (Figures 3C, D). These results indicate that neutrophil infiltration in the heart was reduced after Nav treatment for MI, thereby reducing cardiac damage and remodeling. Additionally, we have included immunofluorescence staining for CD45^+^ cells in Figure 3E to better visualize the inflammatory cells in the treated tissues. Our experiments show a reduction in CD45^+^ immune cells following Navarixin treatment, which further supports its selective effect on neutrophils and provides a more comprehensive understanding of its anti-inflammatory mechanism.

### 3.4 Nav treatment attenuated the expression of inflammatory cytokines caused by MI and reduced the activation of fibrosis

**FIGURE 4:**
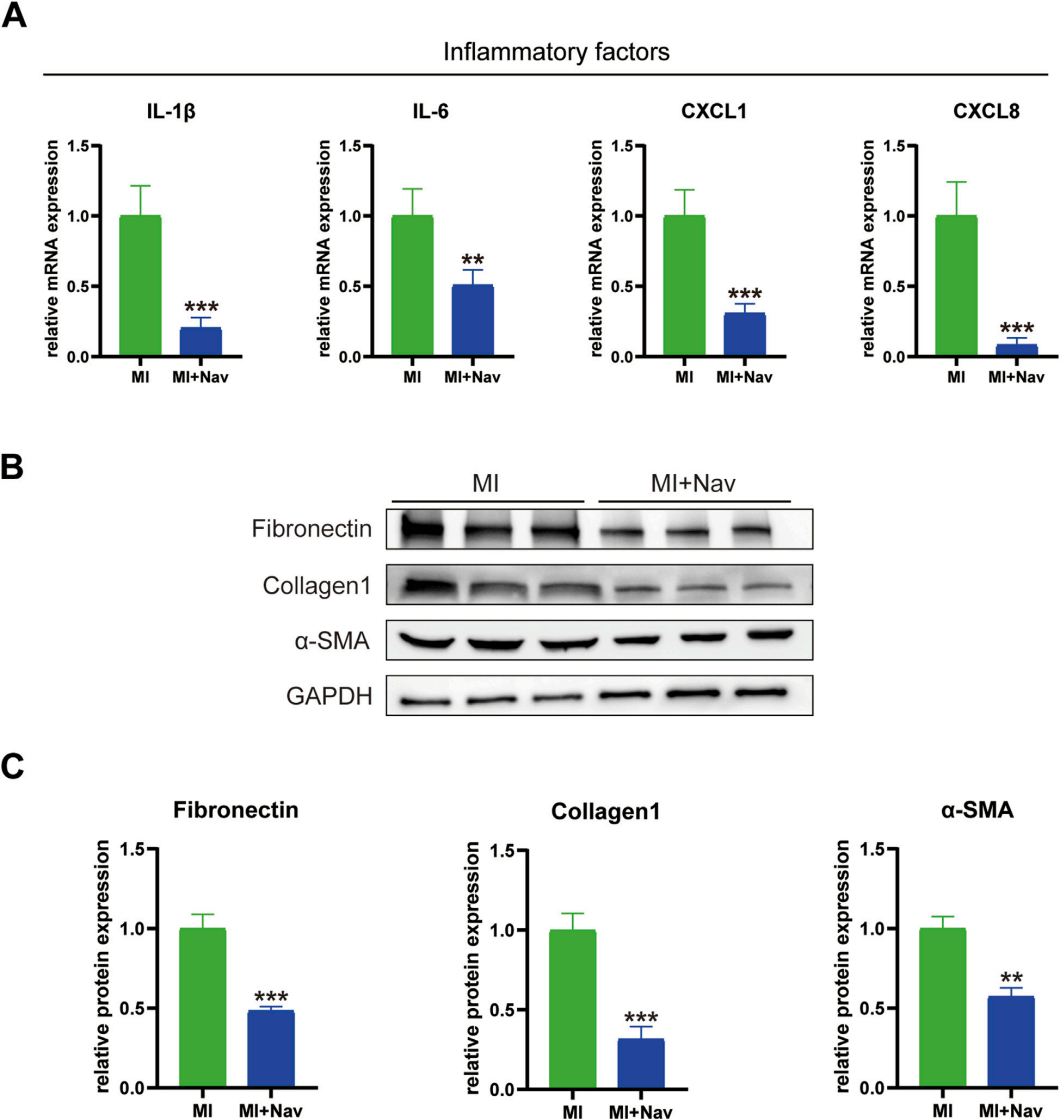
**(A)** mRNA expression levels of the inflammatory factors IL-1β, IL-6, CXCL1 and CXCL8. **(B)** Western blot images of fibronectin, collagen 1, α-SMA and GAPDH expression. **(D–F)** Statistical analysis of the Western blotting results. *P < 0.05, **P < 0.01, ***P < 0.001 vs. the MI group.

To investigate the inhibitory effect of Nav on the inflammatory response to myocardial I/R in mice, we used RT‒PCR to measure the levels of the inflammation-related markers IL-1β, IL-6, CXCL1 and CXCL8 in mouse heart tissue, and the results showed that the expression of these inflammatory factors in the heart tissue of mice treated with Nav was significantly lower than that in the MI group (Figure 4A). If no preventive intervention for myocardial fibrosis is carried out after MI, hyperfibrosis is likely to occur, aggravating the degree of preexisting myocardial fibrosis. With the continuous prolongation of myocardial fibrosis, abnormal cardiac function is not effectively treated, and this increases the probability of MI recurrence. Previous studies have shown that neutrophils can release cytokines and activate the TGF-β pathway in fibroblasts, thereby aggravating cardiac fibrosis. To further confirm the alleviating effect of Nav on cardiac fibrosis, we evaluated the expression of fibrosis marker genes (fibronectin, collagen-1 and α-SMA) and found that the expression of fibrosis marker genes in the infarct boundary zone was reduced after Nav treatment (Figures 4B, C).

### 3.5 Transcriptomic analysis of Nav treatment after MI

**FIGURE 5:**
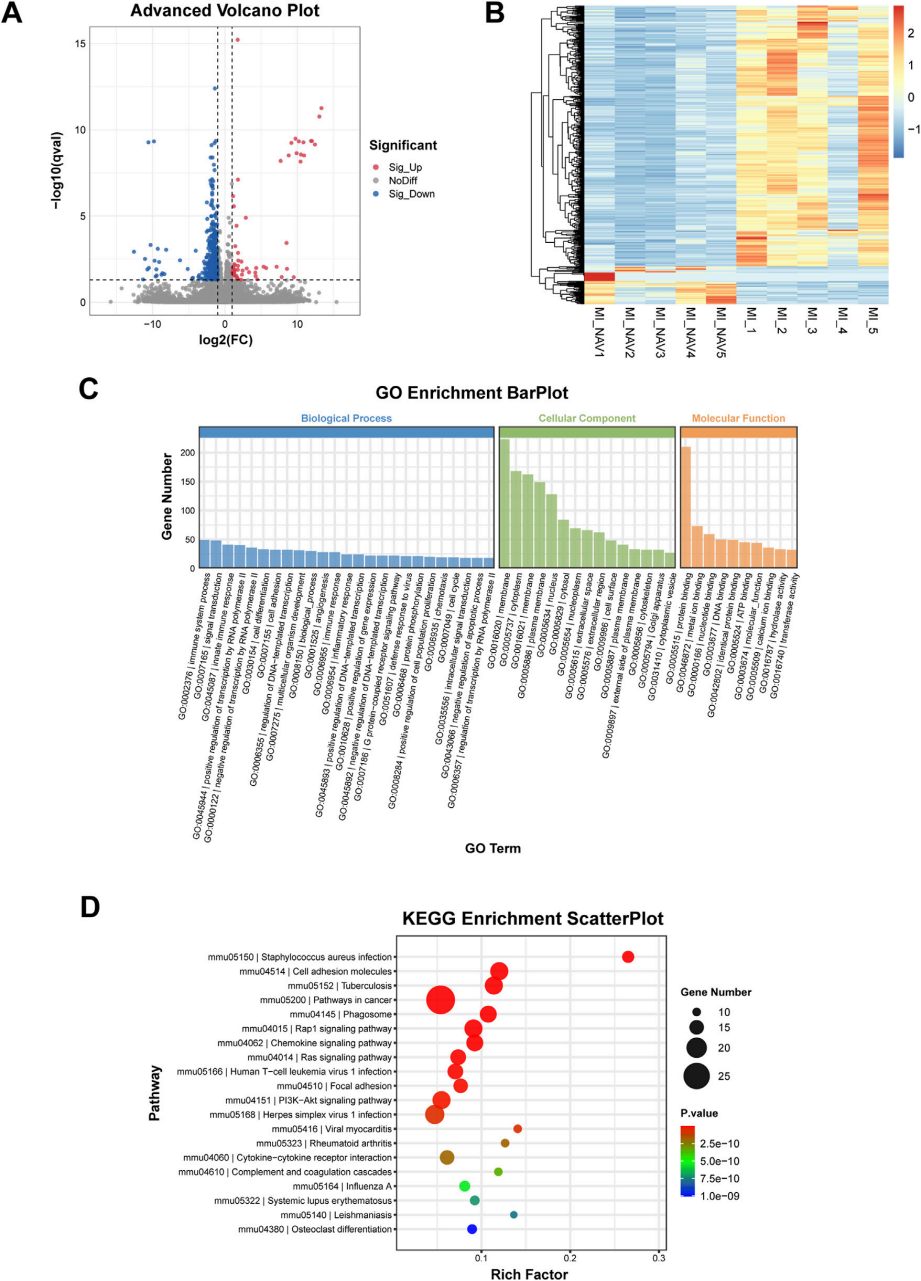
Results of transcriptome sequencing. **(A)** Volcano plot of the differentially expressed genes. **(B)** Differential gene clustering heatmap. **(C)** The histogram of GO enrichment and classification. **(D)** Bubble diagram of the KEGG enrichment results.

To clarify the mechanism by which Nav alleviates ventricular remodeling after MI by reducing neutrophil infiltration, we performed transcriptomic testing on the hearts of the MI and MI + Nav groups of mice. The transcriptome sequencing results are shown in Figure 5, and the number of genes with downregulated expression in the Nav group was significantly greater than the number of upregulated genes. Using (FDR < 0.05 |log2FC| > 1) as the screening criterion, all DEGs were screened, and it was found that the expression of 490 genes in the Nav group changed significantly, 62 genes of which were significantly upregulated and 428 genes of which were significantly downregulated. Stratified cluster analysis of differentially expressed genes was performed, and the results were used to construct a heatmap. GO enrichment and classification of significantly differentially expressed genes (DEGs) revealed significant changes in pathways related to immune system processes and the innate immune response, which are related to reduced neutrophil infiltration. In addition, through KEGG pathway analysis, we found that the chemokine signaling pathway and cytokine‒cytokine receptor interaction were among the top differentially expressed pathways. This finding suggested that Nav affects chemokine-induced immune cell infiltration by inhibiting the CXCR2 receptor. These results corroborate the previously observed decrease in neutrophil infiltration and provide mechanistic support for this finding.

## 4 Discussion

Neutrophils are derived from the bone marrow and are a functionally and phenotypically heterogeneous population of innate immune cells. Neutrophil counts are directly associated with many cardiovascular risk factors, including hypertension and type 2 diabetes, and neutrophilia is associated with the subsequent development of chronic heart failure after acute MI (Horckmans et al., 2017; Dölling et al., 2022; Sreejit et al., 2022). Innate immune cells are involved in cardiomyocyte repair; neutrophils are recruited by chemokines, and they slowly infiltrate the infarcted area within 12–24 h, peaking 2–4 days after MI. When myocardial I/R occurs, multiple innate immune pathways, the complement system, and ROS are activated.

After myocardial ischemia occurs, early reperfusion can significantly reduce the extent of MI and improve cardiac function to some extent. However, after a period of reperfusion, the aggregation of polymorphonuclear leukocytes can lead to myocardial injury through the release of oxygen-derived free radicals, proteases, and leukotrienes. For example, infiltrating neutrophils produce potent cytotoxic effects by releasing proteolytic enzymes, inducing the apoptosis of inflammatory cells. Activated neutrophils drive inflammation and myocardial damage by releasing proinflammatory factors (e.g., IL-1β and IL-6), which interact with the myocardium to degrade the extracellular matrix by releasing matrix metalloproteinases (MMPs) and myeloperoxidase (MPO), producing reactive oxygen species (ROS) and resulting in irreversible myocardial damage (Aul et al., 2012; Kaur and Singh, 2013). Neutrophils can block capillaries, and microvascular embolism leads to a total lack of reflow and secondary ischemia, preventing blood reperfusion and resulting in tissue necrosis and an increased immune response.

The chemokine receptor CXCR2 synergistically binds to its ligands CXCL1-3 and CXCL5-8 to control the release of neutrophils from the bone marrow and recruit them to inflamed and infected tissues. CXCR2 mediates the occurrence and development of various diseases, such as atherosclerosis, myocardial infarction, myocarditis, heart failure and myocardial hypertrophy, and the degree of tissue damage after ischemia is closely related to the number of neutrophils recruited into the tissue (Chapman et al., 2007; Planagumà et al., 2015; Liang et al., 2017). Navarixin functions as a selective CXCR2 antagonist by binding to the extracellular domain of CXCR2, thereby preventing the activation of downstream signaling pathways triggered by the binding of its natural ligands, such as IL-8 and Gro-α. By inhibiting CXCR2 signaling, Navarixin effectively suppresses neutrophil migration and pro-inflammatory cytokine release, which are key processes involved in various inflammatory diseases. Regarding specificity, Navarixin has been demonstrated to be highly selective for CXCR2, with minimal activity against other chemokine receptors, such as CXCR1 and CXCR3. While some off-target effects cannot be fully ruled out, the available data suggest that its binding affinity for CXCR2 is significantly higher compared to other receptors.

In the present study, we utilized the CXCR2 receptor inhibitor Nav to slow post-MI ventricular remodeling by reducing post-MI neutrophil recruitment. We found that Nav administration can alleviate the decline in cardiac function, reduce neutrophil infiltration, reduce the area of cardiac fibrosis, and reduce inflammatory factor expression. Through transcriptomic analysis, we found that Nav affects signaling pathways such as the innate immune response and chemokine signaling pathways.

In the innate immune response pathway, neutrophils have strong phagocytic activity and are the most rapidly mobilized innate immune cells involved in anti-infection processes. They can identify, absorb, and destroy pathogens in the absence of adaptive immune response assistance. Phagocytic engulfment of invading pathogens is a conserved innate immune mechanism. In the chemokine signaling pathway, CXCL8 was the most effective and had the highest cell yield among the seven neutrophil chemokines CXCL1-3 and CXCL5-8. Immobilizing CXCL8 expression on GAGs/proteoglycans and interacting with CXCR2 to induce migration is the key to neutrophil recruitment. Therefore, both of these signaling pathways that cause alterations after Nav treatment are related to the reduction in neutrophil infiltration by antagonism of the CXCR2 receptor.

## 5 Conclusion

In summary, we constructed a mouse model of MI and administered the CXCR2 receptor antagonist Nav. We observed that Nav improved cardiac function, reduced myocardial damage, reduced neutrophil infiltration, reduced inflammatory factor expression and improved cardiac fibrosis in mice. Through transcriptomic analysis, we found that Nav influences signaling pathways such as the innate immune response and chemokine signaling pathways to modulate their effects. By studying and exploring the role and mechanism of the CXCR2 inhibitor Nav in MI-induced heart failure, we provide an experimental basis for CXCR2 inhibitors becoming a new therapeutic target for MI in the future.

## Data Availability

The data presented in the study are deposited in the NCBI (GEO) repository, accession number is GSE291292.
